# Autophagy Pathway Is Required for IL-6 Induced Neuroendocrine Differentiation and Chemoresistance of Prostate Cancer LNCaP Cells

**DOI:** 10.1371/journal.pone.0088556

**Published:** 2014-02-14

**Authors:** Pei-Ching Chang, Tao-Yeuan Wang, Yi-Ting Chang, Cheng-Ying Chu, Chin-Ling Lee, Hung-Wei Hsu, Tyng-An Zhou, Zhaoju Wu, Randie H. Kim, Sonal J. Desai, Shangqin Liu, Hsing-Jien Kung

**Affiliations:** 1 Institute of Microbiology and Immunology, National Yang-Ming University, Taipei, Taiwan, R.O.C; 2 Department of Pathology, Mackay Medical College and Mackay Memorial Hospital, New Taipei City, Taiwan, R.O.C; 3 Mackay Junior College of Medicine, Nursing, and Management, New Taipei City, Taiwan, R.O.C; 4 Institute for Translational Medicine, College of Medical Science and Technology, Taipei Medical University, Taipei, Taiwan, R.O.C; 5 Department of Biochemistry and Molecular Medicine, University of California Davis, Davis, California, United States of America; 6 UC Davis Cancer Center, University of California Davis, Davis, California, United States of America; 7 Department of Dermatology, New York University School of Medicine, New York, New York, United States of America; 8 Department of Hematlogy, Zhongnan Hospital of Wuhan University, Wuhan, China; 9 Division of Molecular and Genomic Medicine, National Health Research Institutes, Zhunan, Miaoli County, Taiwan, R.O.C; II Università di Napoli, Italy

## Abstract

Prostate cancer (PCa) cells undergoing neuroendocrine differentiation (NED) are clinically relevant to the development of relapsed castration-resistant PCa. Increasing evidences show that autophagy involves in the development of neuroendocrine (NE) tumors, including PCa. To clarify the effect of autophagy on NED, androgen-sensitive PCa LNCaP cells were examined. Treatment of LNCaP cells with IL-6 resulted in an induction of autophagy. In the absence of androgen, IL-6 caused an even stronger activation of autophagy. Similar result was identified in NED induction. Inhibition of autophagy with chloroquine (CQ) markedly decreased NED. This observation was confirmed by beclin1 and Atg5 silencing experiments. Further supporting the role of autophagy in NED, we found that LC3 was up-regulated in PCa tissue that had relapsed after androgen-deprivation therapy when compared with their primary tumor counterpart. LC3 staining in relapsed PCa tissue showed punctate pattern similar to the staining of chromogranin A (CgA), a marker for NED cells. Moreover, autophagy inhibition induced the apoptosis of IL-6 induced NE differentiated PCa cells. Consistently, inhibition of autophagy by knockdown of beclin1 or Atg5 sensitized NE differentiated LNCaP cells to etoposide, a chemotherapy drug. To identify the mechanisms, phosphorylation of IL-6 downstream targets was analyzed. An increase in phospho-AMPK and a decrease in phospho-mTOR were found, which implies that IL-6 regulates autophagy through the AMPK/mTOR pathway. Most important to this study is the discovery of REST, a neuronal gene-specific transcriptional repressor that is involved in autophagy activation. REST was down-regulated in IL-6 treatment. Knockdown experiments suggest that REST is critical to NED and autophagy activation by IL-6. Together, our studies imply that autophagy is involved in PCa progression and plays a cytoprotective role when NED is induced in PCa cells by IL-6 treatment. These results reveal the potential of targeting autophagy as part of a combined therapeutic regime for NE tumors.

## Introduction

Prostate cancer (PCa) is a leading cause of cancer mortality in Western countries and its incidence is rapidly increasing in Asia [1]. Androgen-deprivation therapy (ADT) is used for primary and metastatic androgen-dependent PCa [2]. However, 80% to 90% of PCa patients develop castration-resistant tumors within 3 years after successful ADT. Therapeutic treatment of PCa is hampered by such development of a hormone refractory state, whereby hormone therapy fails, resulting in the disease entering into a more aggressive and ultimately fatal stage [3]. One fascinating but understudied feature of hormone refractory PCa is its association with neuroendocrine differentiation (NED) [4]. NED is a process that is observed during ADT [5], [6]. Usually, cells in a tumor undergoing NED show features that are similar to NE cells and these cells are called neuroendocrine-like (NE-like) cells. NE-like cells are non-proliferative, terminally differentiated, and androgen receptor (AR)-negative. They are very difficult to kill because they are refractory to hormone therapy due to lacking the AR; furthermore, they are resistant to conventional chemotherapy, because they do not divide [7]. Moreover, they release a large number of neurokines, chemokines, cytokines and growth factors; this results in an increase in proliferation of any neighboring non-NE PCa cells; this occurs in a paracrine manner during ADT. NE-like cells are likely to be the root causes of hormone- and chemotherapy resistance of castration-resistant PCa and the presence of NE-like cells is correlated with a poor prognosis [7]–[9]. The ability to identify the novel mechanisms underlying the NED of PCa cells and of the therapeutic resistance of NE-like cells will provide new strategies that can be apply to the prevention of relapsed castration-resistant PCa or, alternatively, to the development of combined therapeutic regimes for relapsed castration-resistant PCa.

NE-like cells can be identified based on morphological changes and the expression of neuronal markers. Multiple pathways have been shown to induce NED in PCa cells using *in vitro* culture systems; these include androgen deprivation [10] and interlerukin-6 (IL-6) treatment [11]. The latter is particularly important as IL-6 levels are significantly increased in patients undergoing ADT and clinical studies have demonstrated that the serum levels of IL-6 are frequently higher in patients with castration-resistant and metastatic PCa [12]–[14]. IL-6 is a pleiotropic cytokine important for various immune responses, cell survival, proliferation and tumorigenesis [15], [16]. Canonical IL-6 signaling pathways include (i) JAK-STAT3, (ii) PIK3-Akt and (iii) MEK-ERK. Studies have demonstrated that IL-6 mediates growth arrest and induces NED in PCa cells via the activation of distinctive signaling pathways; these include STAT3 [17] and PIK3-Etk/Bmx [18]. Recently, Delk *et al* showed that IL-6 secreted by bone marrow stromal cells induced NED and autophagy in bone metastatic PCa cells through an STAT3-independent pathway [19]. Thus, IL-6 has been suggested to induce NED and facilitated PCa cells becoming refractory. This makes IL-6 an attractive target for therapy. However, due to its pleiotropism, targeting IL-6 is likely to result in unpredictable responses. An improved understanding of the cellular events associated with IL-6 exposure may help identify potential effective target(s) for the prevention and/or treatment of PCa.

Autophagy, also called macroautophagy, is a major regulated lysosomal degradation pathway that eukaryotic cells use to degrade long-lived proteins and organelles in response to nutrition starvation or metabolic stress. This self-digestion pathway is essential for normal development and for maintaining the intracellular metabolic homeostasis of terminally differentiated cells such as neurons. It is also a central biological pathway that functions to protect organisms against various pathogens and against cancer; it thus is able to promote health and longevity. However, when autophagy is hijacked by cancer cells, it emerges as a way to protect tumor cells from dying, and hence is able to confer drug-resistance on the cancer cells. Understanding of the molecular mechanisms associated with autophagy began in 1993 when autophagy-related genes (Atgs) were first identified in *S. cerevisiae* [20]. Autophagy is a multi-step process and thus can be divided into several stages; (i) induction, (ii) vesicle nucleation, (iii) vesicle elongation and completion to form the autophagosome, (iv) docking and fusion with lysosome to form autolysosome, (v) degradation, and (vi) retrieval [21]. Among the main upstream regulators of the autophagic pathway, the class I PI3K (PI3KI)-Akt [22] and MEK1/Erk [23], [24] molecules link receptor tyrosine kinases to mTOR; these act as key negative regulators of autophagy and thereby repress autophagy in the presence of insulin-like and other growth factor signals. Interestingly, the function of class III PI3K (PI3KIII) is completely opposite to that of PI3KI when regulating autophagy. By interacting with the core component beclin1, PI3KIII accelerates autophagy by promoting vesicle nucleation [25]. One minor mechanisms involves LKB1-AMPK molecules, which link the intracellular energy status of cells to the negative regulation of mTOR; this pathway acts to activate autophagy in the presence of stresses that increase the AMP/ATP ratio [26]. Other minor mechanisms include p53, ER stress-Ca^2+^ signaling, and cytoplasmic STAT3. The role of p53 in autophagy is paradoxical and depends on its subcellular location [27]–[29]. ER stress accompanying Ca^2+^ signaling is also involved in the regulation of autophagy and this occurs by activating CaMKKβ-dependent AMPK pathway [30], [31]. Finally, cytoplasmic STAT3 suppresses autophagy by binding to protein kinase R (PKR) and inhibiting the phosphorylation of eIF2α [32].

NE-like cell development relies on a network of transcriptional repressors and activators that controls the acquisition and maintenance of neuronal features. Repressor element-1 silencing transcription factor/neuron restrictive silencing factor (REST/NRSF) was first discovered as a master transcriptional repressor that is responsible for restricting neuronal gene expression in non-neuronal cells [33]–[36]. The repressor binds to a 21-bp repressor element 1/neuron-restrictive silencing element (RE1/NRSE) and then recruits a repressive chromatin remodeling complex, mSin3 and Co-REST. This occurs via its two repression domain (one at the amino terminus and the other at the carboxyl terminal) and the result is the epigenetic silencing of the transcription of the target gene [37]. REST is highly expressed in embryonic stem cells (ESCs), neural progenitor cells (NPCs) and non-neuronal cells, where the protein suppresses the expression of neural specific genes, thus maintaining pluripotency of ESCs and NPCs by inhibiting neuronal differentiation in non-neuronal cells [33]. Interestingly, REST has been demonstrated to inhibit the gene transcription of synaptophysin (SYN) [38], [39], one of the common markers of NED in PCa cells. A very recent report by Svensson *et al* also showed that REST mediates AR actions and modulates androgen-deprivation induced NED in PCa [40].

In order to target the contribution of NED to the progression of PCa into the hormone-refractory state, the present study has the aim of determining whether IL-6 is able to up-regulate autophagy in PCa cells, which, in turn, induces cell NED. This will prevent cell apoptosis during IL-6 induced NED and as a result will allow NE-like cell survival in relapsed PCa. In order to address our hypothesis, we investigated the ability of IL-6 to induce autophagy and NED in androgen-sensitive LNCaP cells. We found that autophagy is induced by IL-6 and plays an essential role in IL-6 induced NED. Consistent with the activation of autophagy during NED, an increased level of LC3 can be observed in relapsed PCa tissue and such tissue has a similar foci staining pattern for CgA, a marker for NE-like cells. We investigated autophagy as a potential signal that is able to protect PCa cells from apoptosis and the chemotherapy drugs such as etoposide. Consistent with the increased level of autophagy, AMPK activation and mTOR inhibition was demonstrated to be present during IL-6 treatment in the absence of androgen. Most importantly, our study identified the down-regulation of REST, a neuron-restrictive silencer; this was found to be critical to NED induction and autophagy activation by IL-6.

## Materials and Methods

### Cell Culture and Plasmids

LNCaP PCa cells were cultured in RPMI 1640 (Gibco/Invitrogen, 31800–014) containing 10% fetal bovine serum (FBS) (Hyclone, SH30071.03), 1% penicillin/streptomycin (Sigma-Aldrich, P4458) and L-glutamine (Sigma-Aldrich, G7513). LNCaP cells stably expressing eGFP-LC3, LNCaP-eGFP-LC3, had been generated previously [41] and were maintained as described for LNCaP, but supplemented with 400 µg/mL of G418 (Amresco, E859). The inducible shRNA cassette, the H1/TO-shRNA-linker containing a BsmBI cutting site, was introduced into the pLenti4 vector in order to generate an inducible shRNA lentiviral vector, namely plenti4-H1/TO-shRNA. The inducible promoter for shRNA expression is a hybrid of the H1 promoter and the tet operator (Invitrogen, H1/TO). The shRNA cassette of beclin1 (5′-CACCGGTCTAAGACGTCCAACAACACGAA TGTTGTTGGACGTCTTAGACC-3′), Atg5 (5′-CACCAAGCAACTCTGGATGGG ATTGCGAACAATCCCATCCAGAGTTGCTT-3′) and REST (5′-CACCGTGTAA TCTACAGTATCACCGAAGTGATACTGTAGATTACAC-3′) were individually inserted into the plenti4-H1/TO-shRNA and these were then introduced into LNCaP-TR cells by lentiviral transduction. The cells were selected for 14 days using 50 µg/ml zeocine (InvivoGen, ant-zn-1). The knockdown efficiencies for beclin1, Atg5 and REST by the shRNAs were tested by being treated with doxycycline (Dox) for 48 hours. LNCaP-TR-shBeclin1, LNCaP-TR-shAtg5, LNCaP-TR-shREST were maintained as described for LNCaP, but supplemented with 5 µg/ml of blasticidin S (InvivoGen, ant-bl-1) and 50 µg/ml of zeocine.

### Fluorescent Microscopy

LNCaP cells were plated on poly-L-lysine-coated coverslips (Marienfeld, 0111530) and after being treated as indicated, they were fixed with 4% paraformaldehyde in 1× PBS for 15 minutes. Next the coverslips were mounted in mounting solution [20 mM n-propylgallate, 80% Glycerol, 20% 1×PBS], and visualized by phase-contrast microscopy (Lecia, DMI4000B). The length of the neurites on the coverslips was analyzed by MetaMorph (Molecular Devices, Neurite Outgrowth). LNCaP cells show low basic level of NED under normal culture condition (RPMI 1640 supplemented with 10% FBS). The average neurite length of control cells cultured in RPMI 1640 containing 10% FBS in each experiments was used as 1, and a comparison was then made for neurite length obtained after IL-6 treatments. The LNCaP-eGFP-LC3 cells were prepared as described above for LNCaP cells except for staining with Hochest 33258 (Invitrogen, H3569) before mounting. The eGFP-LC3 images were visualized using a Lecia DMI4000B fluorescence microscope with a 63× lens and were analyzed by MetaMorph (Molecular Devices, Transflour). LNCaP cells show low basic level of autophagy under normal culture condition. To distinguish the autophagy induction, cells displaying more than 50 intense eGFP-LC3 aggregates of 5 to 7.5 µm and 15 intense eGFP-LC3 aggregates of 7.5 to 20 µm were counted as autophagy-positive cells after induction.

### Western Blot and Antibodies

Total cell lysates (TCLs) were prepared from cells using NP-40 lysis buffer [0.5% NP-40 (Amresco, E109), 1× PBS, 1× protease inhibitors (Roche, 04693132001)]. Protein concentration of each sample was then measured using Bio-Rad protein assay dye reagent and the manufacturer's protocol (Bio-Rad, 500–0006). For immunoblotting, equal protein amounts were loaded onto and separated on a 6%, 8% or 15% SDS-polyacrylamide gel as appropriate. The separated proteins were then transferred from the gel to 0.45 µm pore size PVDF membranes (GE Healthcare, RPN303F). Next the membranes were blocked with blocking buffer [5% BSA in 1× TBST]. Primary antibodies for p-Akt (Ser473) (Cell signaling, #9271), Akt (Cell signaling, #9272), p-mTOR (Ser2248) (Cell signaling, #2976), mTOR (Cell signaling, #2983), p-STAT3 (Tyr705) (Cell signaling, #9145), STAT3 (Cell signaling, #9139), p-Erk (Thr202/Tyr204) (Cell signaling, #9101), Erk (Santa Cruz Biotechnology, sc154), p-AMPK (Thr172) (Cell signaling, #2535), AMPK (Cell signaling, #2793), LC3B (Cell signaling, #2775), beclin1 (Cell signaling, #3738), Atg5 (Cell signaling, #2630), tubulin III (Cell signaling, #5666), androgen receptor (Millipore, 06–680), REST (Millipore, 09–019), and GAPDH (GeneTex, GTX100118) were diluted in 5% BSA in 1× TBST. The membranes were then probed with the various primary antibodies, then treated with the appropriate secondary antibody. Finally, the membranes were visualized using a Pierce ECL Western Blotting Substrate (Thermo Scientific, 34080) and imaged via a Luminescence/Fluorescence Imaging System (FUJIFILM, LAS-4000).

### Immunohistochemistry Staining (IHC-staining)

Paraffin-embedded specimens from patients with primary and relapsed castration-resistant PCa that had been collected between 1990 and 2010 at Mackay Memorial Hospital were included in this study. Ethics was approved by the Mackay Memorial Hospital Institute Review Board. Informed consent was written. Tissue sections were deparaffinized in xylene, rehydrated through graded ethanol (100, 90, 80, 70, 50%), subjected to antigen retrieval by microwaving in 10 mM citrate buffer (pH 6.0) for 10 min, blocked for endogenous peroxidase activity with 3% H_2_O_2_ for 10 min at room temperature, and then blocked with antibody diluent containing background-reducing components (DAKO, S302281). Finally, the slides were stained overnight at 4°C with beclin1 (GeneTex, GTX61619), LC3 (Novus Biologicals, NB110-57179), REST (BETHYL, IHC-00141), or CgA (abcam, ab15160) specific antibodies and visualized following standard protocol using 3-amino-9-ethylcarbazole (AEC) as chromogen (DAKO, K346430) in the presence of hematoxylin counterstaining. The histology and staining was evaluated by a pathologist in a blind fashion using a four-tier scale representing negative (0), weak (1), intermediate (2), and strong (3) signals.

### The 3-(4,5-Dimethylthiazol-2-yl)-2,5-diphenyltetrazolium bromide (MTT) Assay

LNCaP, LNCaP-TR-shBeclin1, and LNCaP-TR-shAtg5 cells were seeded into 96-well plates, cultured for two days, and then treated as indicated. Next, the cell viability was examined by MTT assay (Sigma-Aldrich, M5655). MTT was added at a final concentration of 0.5 mg/mL and the formazan crystals were solubilized by 10% SDS. The optical density (OD) was quantified using a microplate spectrophotometer at a wavelength of 570 and 660 nm.

### The Caspase-3/7 ELISA Assay

LNCaP cells were plated into 96-well plates. The following day, the cells were treated as indicated. Caspase-3/7 activity levels were then measured using a Apo-ONE Homogeneous Caspase-3/7 Assay kit (Promega, TB295) according to the manufacturer’s protocol. The cells were incubated for 30 min at room temperature with the mixture of substrate and reaction buffer. The caspase-3/7 activities were then measured using a fluorescence microplate reader (Tecan Systems, Infinite 200).

### Reverse Transcription (RT) and Quantitative PCR (qPCR)

Total RNA was isolated from non-induced and Dox-induced LNCaP-TR-shREST cells using TRIzol reagent (Invitrogen, 15596-018). Total RNA (2 µg) was reverse transcribed using Oligo-dT and SuperScript^III^ RT (Invitrogen, 18080-085). The mRNA levels of various genes were then determined by qPCR and normalized against GAPDH.

## Results

### IL-6 and Androgen Deprivation Synergistically Induce Autophagy in LNCaP Cells

**Figure 1:**
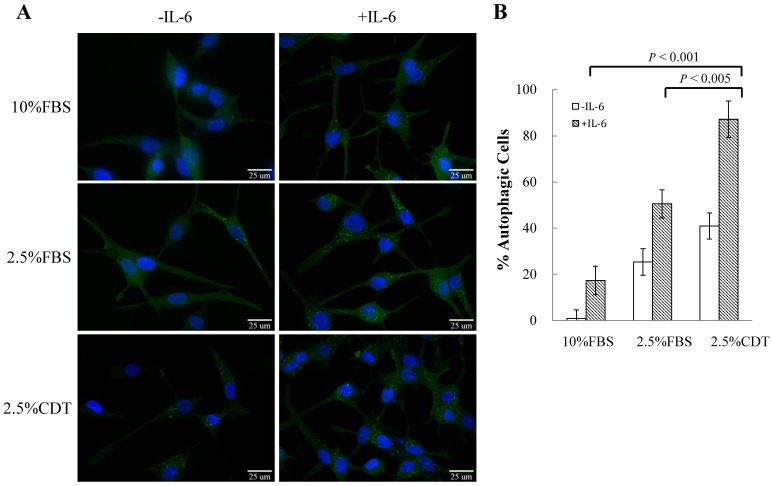
The potentiating effect of androgen-deprivation on IL-6-induced autophagy in LNCaP cells. (A) LNCaP-eGFP-LC3 cells were culture in 10% FBS, 2.5% FBS or 2.5% CDT supplemented RPMI 1640 in the absence (control) and presence of 100 ng/ml IL-6 for 48 hours. This was followed by fixation, nuclear counterstaining with DAPI (blue), and analysis by fluorescence microscopy (FITC, 63× magnification). (B) For each treatment, the percentage of cells with eGFP-LC3 punctate was calculated using the average from 15 microscopic fields; *bars*, SD.

**Figure 2:**
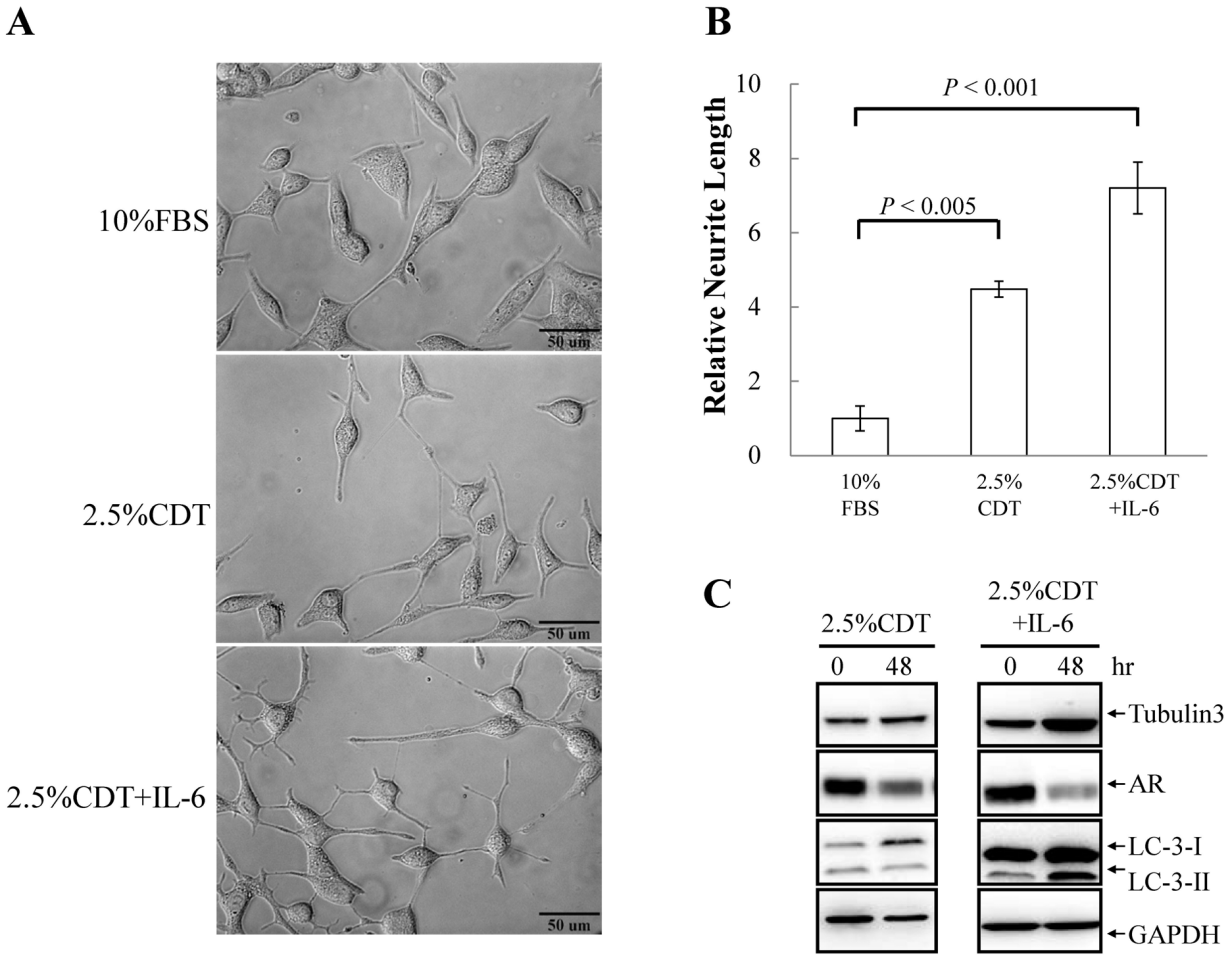
IL-6 induces NED in LNCaP cells and this is concomitant with increased autophagy. (A) LNCaP cells were treated with 2.5% CDT or 2.5% CDT plus 100 ng/ml IL-6 for 48 hours. The induced neurite elongation was assessed using brightfield microscopy images (40× magnification). (B) The neurite elongation was quantified using the average from 3–5 microscopic fields; *bars*, SD. (C) LNCaP cells were treated as described in (A). Total cell lysates (TCLs) were prepared and then immunoblotted to detect tubulin III, androgen receptor (AR) and LC3. GAPDH was used as the loading control.

Increasing evidence has shown that autophagy plays an important role in supporting neuronal differentiation [42]–[44]. A more recent study also shows that antophagy may play an essential role in IL-6 induced NED of bone metastatic PCa cells [19]. The serum levels of IL-6 have been found to increase in patients undergoing androgen-deprivation therapy (ADT) [12], [14]. Androgen-deprivation [45] and IL-6 [18] have also been found responsible for the NED development and PCa refractory to ADT. To study whether autophagy is activated along with NED of PCa cell during ADT, androgen deprivation and IL-6 combined treatment were used in here. LNCaP cell line, an androgen-sensitive human prostate adenocarcinoma cell line rapidly acquires NE characteristics, including cessation of mitosis, neurite extension and enhanced expression of neuronal markers such as tubulin III, when stimulated, was chosen for the study. LNCaP cells stably expressing eGFP-LC3, namely LNCaP-eGFP-LC3 cells, were cultured in regular medium containing 10% FBS, 2.5% *charcoal/dextran*-treated FBS (*CDT*) (the androgen-deprivation condition), or 2.5% CDT plus 100 ng/ml IL-6. Autophagosomic cells (cells with GFP-LC3-II punctation) were count using fixed LNCaP cells after 48 hours of treatment. Androgen-deprivation increased the number of cells undergoing autophagy to 41% (Fig. 1). IL-6 alone increased the number of autophagic cells to 17% (Fig. 1). Interestingly, the number of cells undergoing autophagy induced by IL-6 under androgen-deprivation was significantly enhanced further to 87% (Fig. 1). The induction of NED by androgen deprivation and IL-6 treatment was confirmed using the LNCaP cells. Neurite elongation, a typical phenotype of NED cells, was observed (Fig. 2A and 2B). Intriguingly, the degree of autophagy induction was highly correlated with the degree of NED among LNCaP cells. This correlation was further confirmed by Western blot analysis. IL-6 treatment significantly increased the expression of the neuron-specific marker, tubulin III and the conversion of LC3-I to LC3-II (Fig. 2C). Our findings suggest that the activation of autophagy might be required for IL-6-induced NED under androgen deprivation conditions.

### Autophagy Inhibition Suppresses NED in LNCaP Cells

**Figure 3:**
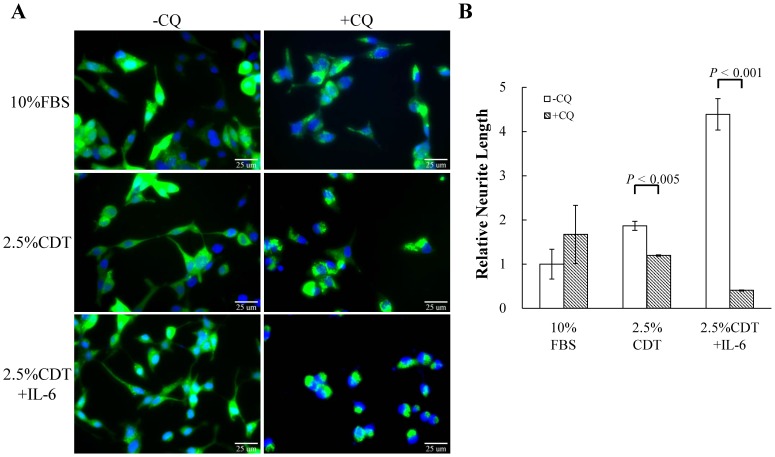
Chemical inhibition of autophagy flux suppresses IL-6-induced NED in LNCaP cells. (A) LNCaP-eGFP-LC3 cells were treated with 2.5% CDT or 2.5% CDT plus 100 ng/ml IL-6, in the absence and presence of 50 µM chloroquine (CQ) for 48 hours. This was followed by fixation, nuclear counterstaining with DAPI (blue), and analysis by fluorescence microscopy (FITC, 40× magnification). (B) The neurite elongation was quantified using the average from 3–5 microscopic fields; *bars*, SD.

**Figure 4:**
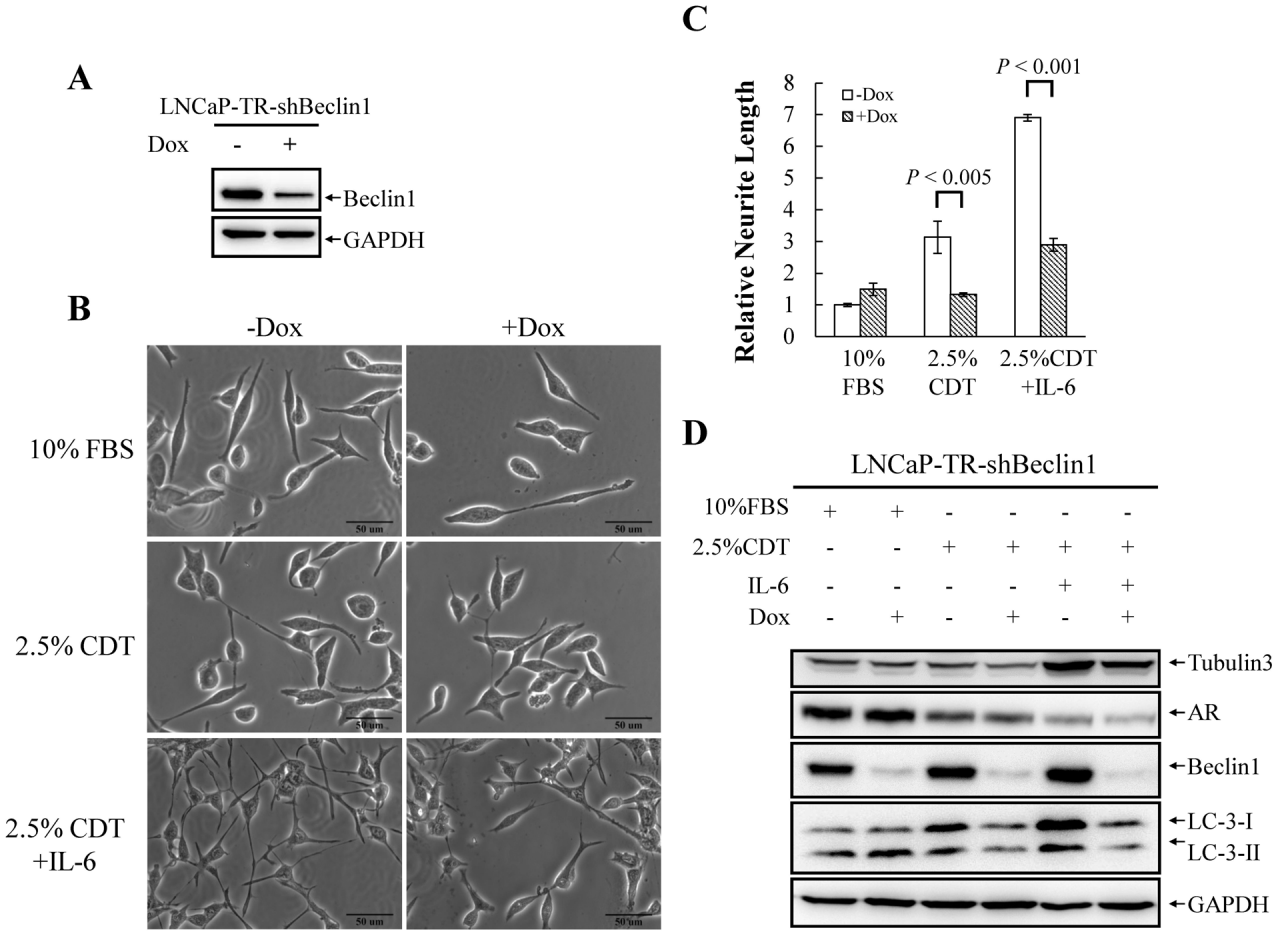
Knockdown of beclin1 suppresses IL-6 induced NED under androgen deprivation conditions. (A) LNCaP-TR-shBeclin1 cells were treated with 1 µg/ml Dox for 48 hours. TCLs were prepared and immunoblotted to detect beclin1 using GAPDH as the loading control. (B) LNCaP-TR-shBeclin1 cells were treated for 48 hours with 1 µg/ml Dox in order to induce knockdown of beclin1 and they were then treated for another 48 hours with 2.5% CDT or 2.5% CDT plus 100 ng/ml IL-6 to induce cell NED. The inhibition of neurite elongation by beclin1 knockdown was assessed using brightfield microscopy images (40× magnification). (C) The neurite elongation was quantified using the average from 3–5 microscopic fields; *bars*, SD. (D) TCLs were obtained from LNCaP-TR-shBeclin1 cells treated as described in (A) and these were then analyzed by immunoblotting using the indicated antibodies.

**Figure 5:**
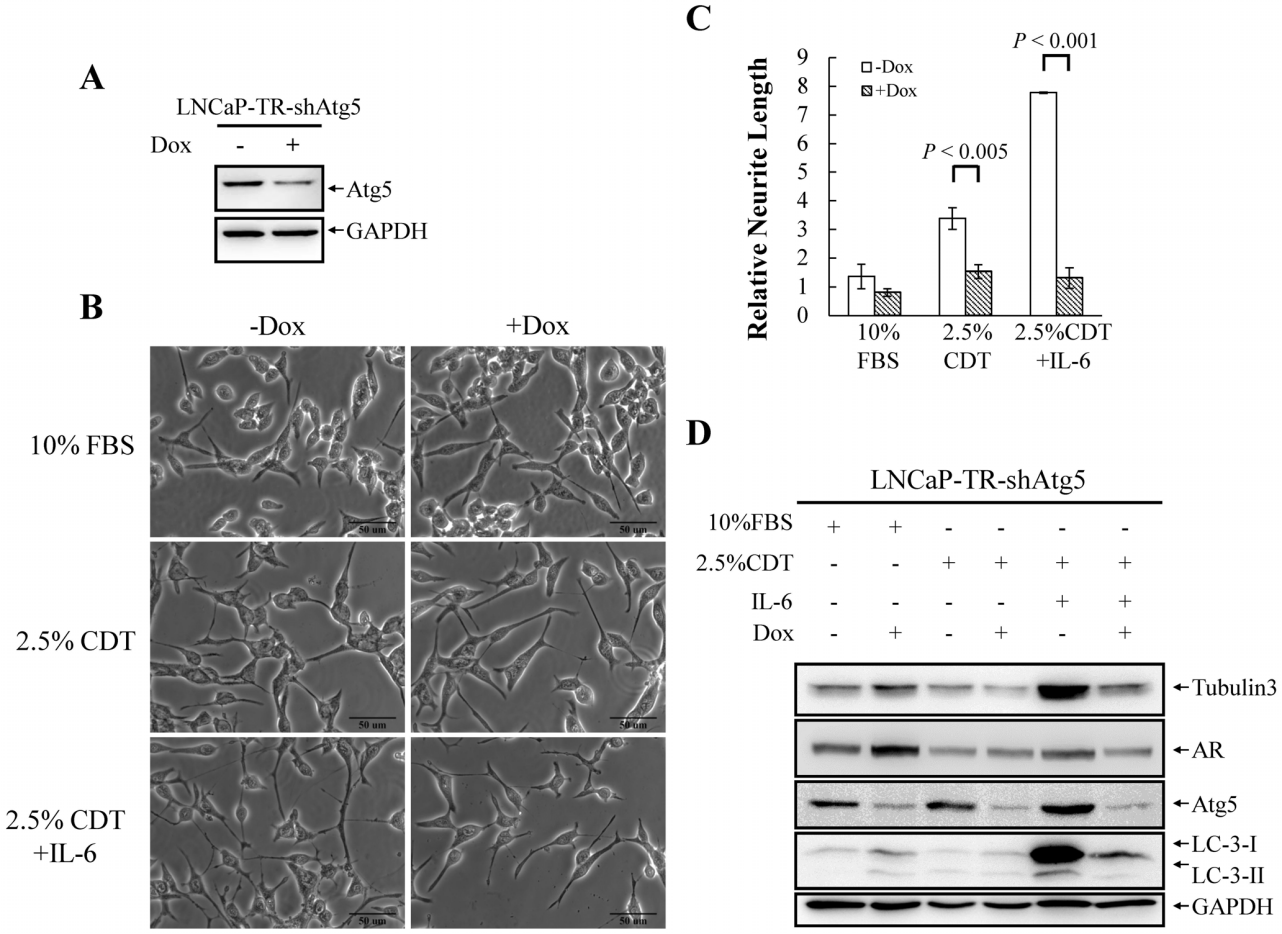
Knockdown of Atg5 suppresses IL-6 induced NED under androgen deprivation conditions. (A) LNCaP-TR-shAtg5 cells were treated with 1 µg/ml Dox for 48 hours. TCLs were prepared and immunoblotted to detect Atg5 using GAPDH as the loading control. (B) LNCaP-TR-shAtg5 cells were treated for 48 hours with 1 µg/ml Dox to induce knockdown of Atg5 and then treated for another 48 hours with 2.5% CDT or 2.5% CDT plus 100 ng/ml IL-6 to induce cell NED. The inhibition of neurite elongation by Atg5 knockdown was assessed using brightfield microscopy images (40× magnification). (C) The neurite elongation was quantified using the average from 3–5 microscopic fields; *bars*, SD. (D) TCLs obtained from LNCaP-TR-shAtg5 cells treated as described in (A) and these were then analyzed by immunoblotting using the indicated antibodies.

To determine whether autophagy induction is essential for IL-6-induced NED under the androgen deprivation conditions, we inhibit autophagy using chloroquine (CQ), an autophagy inhibitor that block the functioning of the lysosome. As shown in Figure 3A, CQ (50 µM) strongly inhibited IL-6-induced NED in LNCaP cells and slightly reduced the differentiation induced by androgen deprivation. Quantification of neurite length by MetaMorph showed there was also significant inhibition of this phenotype (Fig. 3B). CQ may have non-specific effects other than that the inhibition of the autophagy pathway. To further confirm the importance of the autophagy pathway to IL-6-induced NED under the androgen deprivation conditions, we employed small hairpin RNAs (shRNAs), shBeclin1 and shAtg5, to knockdown the expression of beclin1 (Atg6) and Atg5, two Atg genes essential for autophagy initiation and autophagosome formation, respectively. First, we established a shBeclin1 inducible knockdown cell line and a shAtg5 inducible knockdown cell line in LNCaP cells, namely LNCaP-TR-shBeclin1 and -shAtg5. Immunoblotting showed that both shRNAs were able to knockdown their target successfully (Fig. 4A and 5A). Interestingly, beclin1 knockdown cells displayed a significantly lower degree of NED than control cells (Fig. 4B and 4C) and a similar result was observed in Atg5 knockdown cells (Fig. 5B and 5C). Quantification data showed that both Atg5 and beclin1 knockdown has significant inhibition efficiency in IL-6 induced NED (Fig. 4C and 5C). Consistent with the cell morphology, inhibition of NED by knocking down beclin1 and Atg5 was identified by Western blot analysis using tubulin III antibody (Fig. 4D and 5D). Together, these findings demonstrate that the autophagy pathway is essential for PCa cells to undergo NED.

### Autophagy Gene Expression in Primary and Relapsed PCa Specimens

**Figure 6:**
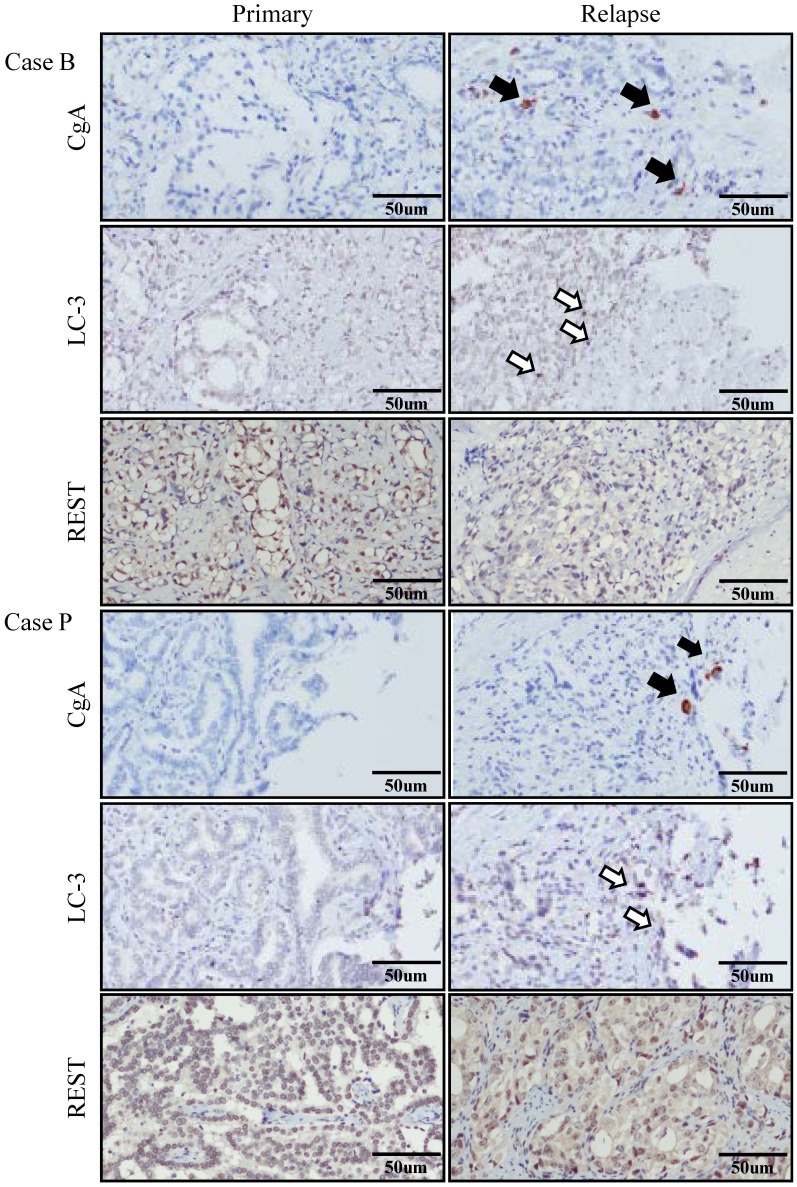
Up-regulation of the autophagy related genes LC3, the NE marker Chromogranin A (CgA), and the down-modulation of REST in relapsed PCa tissue compared to their primary tumor counterpart. Representative IHC staining images of two pairs of tissue samples, namely primary and relapsed PCa specimens from the same individual, were stained using anti-LC3, anti-CgA, and anti-REST antibodies.

The above findings suggest that autophagy may be activated along with NED in PCa cells during hormone-refractory relapse. To characterize the relationship between autophagy and NED in PCa cells, we examined the expression of CgA, which is a NE tumor marker, and the expression of LC3, a autophagy related genes, in thirteen pairs of primary and hormone-refractory relapsed PCa tissue samples, the pairs being obtained from the same patient. Representative immunohistochemistry (IHC) results for CgA and LC3 are shown in Figure 6. The positive CgA staining shows a foci pattern (Fig. 6, closed arrows), which is a typical feature of NE cells in relapsed PCa specimens; however this pattern was not present in the primary PCa specimens. Interestingly, a foci staining of LC3 was also observed in relapsed PCa specimens (Fig. 6, open arrows). Out of the thirteen pairs of PCa specimens, 8 (62%) showed a significant increase in LC3 expression (average LC3 immunoreactivity (IR) of primary and relapsed PCa tumor was 0.51 and 1.12, respectively; P<0.005) in relapsed PCa tissue comparing to their primary tumor counterpart.

### Autophagy Induced by IL-6 Protects NE Differentiated LNCaP Cells from Cell Death

**Figure 7:**
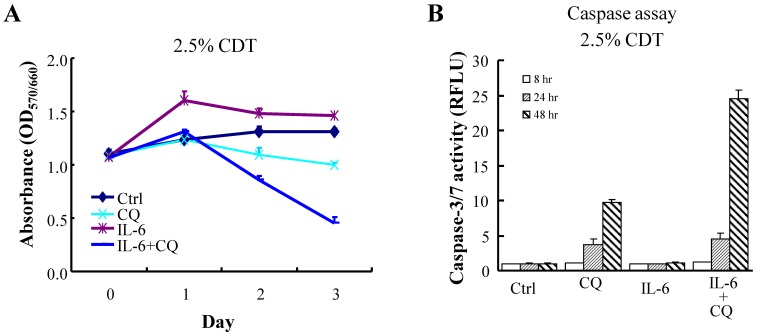
Inhibition of autophagy by CQ results in the cell death of IL-6-induced NE differentiated LNCaP cells. (A) LNCaP cells were cultured in 2.5% CDT supplemented RPMI 1640 and then treated with 100 ng/ml IL-6 or vehicle control in the presence or absence of 50 µM CQ. Cell numbers were analyzed using a MTT cell viability assay at the indicated time points. *Points*, mean; *bars*, SD. (B) LNCaP cells were treated as described in (A) and then assayed for caspase-3/7 activity at the indicated time points. Each data point shown is the mean of three independent experiments; *bars*, SD.

Autophagy is important to the maintenance of homeostasis of terminally differentiated cells [46]. The observation that autophagy is neuroprotective [47] and that the autophagy is induced by IL-6 prompted us to hypothesize that autophagy may serve as a protective mechanism for maintaining homeostasis and increasing the survival of IL-6-induced terminally differentiated NE-like cells. If this is the case, cell killing ought to be potentiated if autophagy is inhibited. To this end, we performed an MTT assay to identify the role of the autophagic pathway in IL-6 induced growth arrest and resistance to cell death. Consistent with previous studies, IL-6 treatment induced growth arrest in LNCaP cells (Fig. 7A). When autophagy was inhibited by treatment with CQ, this induced a significant increase in cell death among IL-6 treated LNCaP cells (Fig. 7A). The increased cell killing was found to be largely due to enhanced apoptosis, as evidenced by the presence of a significant increase in caspase-3/7 activity at 48 hours after IL-6 plus CQ combined treatment (Fig. 7B). These findings show for the first time that inhibition of autophagy is able to overcome the apoptosis-resistance of IL-6-indued NE differentiated cells and indicate that autophagy is the underlying cause behind the chemoresistance of NE-like cells.

**Figure 8:**
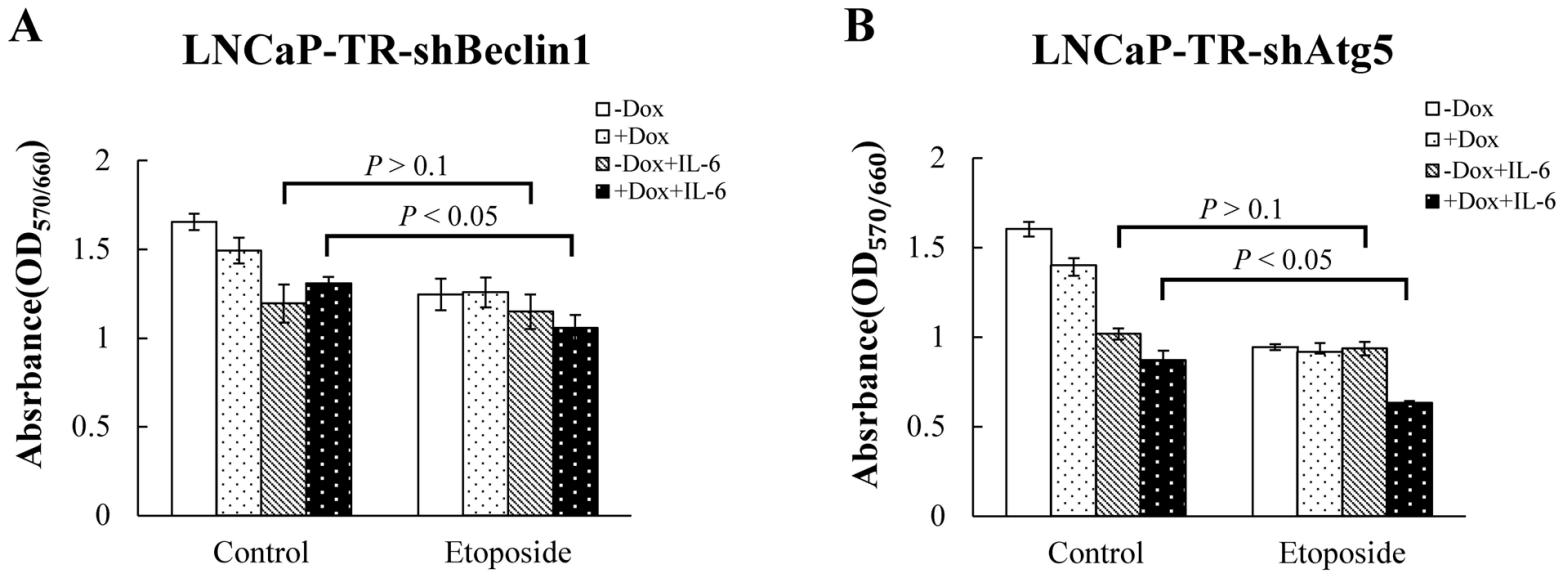
Knockdown of beclin1 or Atg5 independently enhances the sensitivity of IL-6-induced NE differentiated LNCaP cells to etoposide. LNCaP-TR-shBeclin1 (A) and LNCaP-TR-shAtg5 (B) cells were treated with Dox for 48 hours to induced either beclin1 or Atg5 knockdown, respectively, or left untreated. LNCaP control and LNCaP knockdown cells were pre-incubated with 2.5% CDT or 2.5% CDT plus 100 ng/ml IL-6 for 48 hours followed by treatment with 20 µg/ml etoposide for 9 days before the MTT assay. Each data point shown is the mean of three independent experiments; *bars*, SD.

The protective role of autophagy in the chemoresistance of NE differentiated PCa cells to chemotherapeutic drugs such as etoposide was then confirmed by the following findings. As expected, etoposide was unable to kill the IL-6 induced NE differentiated LNCaP cells. Interestingly, etoposide induced a statistically significant increase in cell death after knockdown of either beclin1 or Atg5 in NE differentiated LNCaP cells that had been induced by IL-6 under androgen deprivation conditions (Fig. 8). When taken together, the above findings suggest that autophagy induced by IL-6 is able to protect IL-6-induced NE differentiated LNCaP cells from apoptotic cell death and that an inhibition of autophagy is able to abrogate the apoptosis resistance as well as the chemotherapy resistance associated with NE-like PCa cells.

### mTOR is Inhibited during IL-6 Induces Autophagy Under Androgen Deprivation Conditions

**Figure 9:**
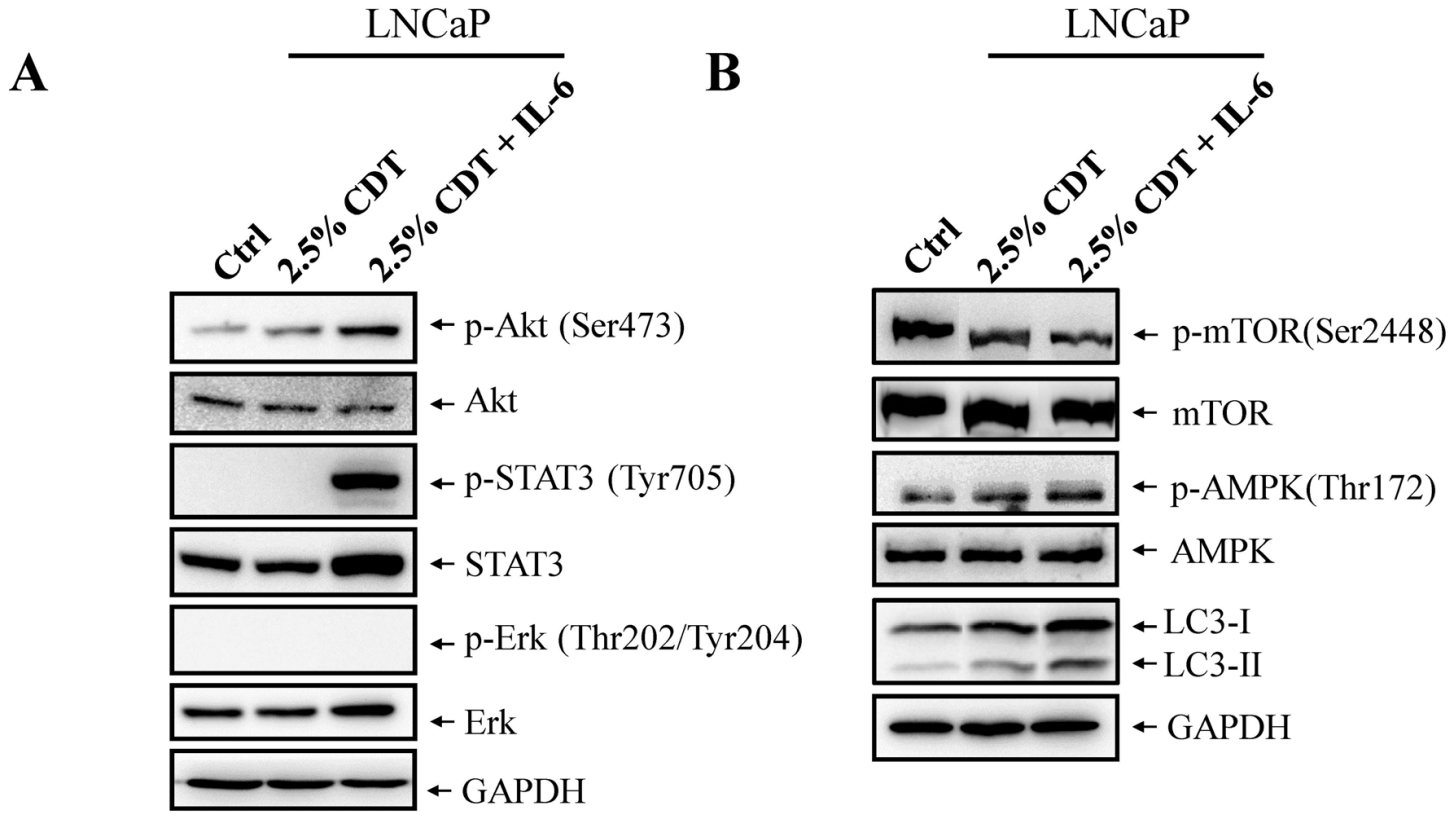
IL-6 treatment inhibits mTOR via the activation of AMPK pathway. (A) LNCaP cells were treated for 48 hours with 2.5% CDT or 2.5% CDT plus 100 ng/ml IL-6. TCLs were prepared and immunoblotted using phospho-STAT3, phospho-Akt and phopho-ERK specific antibodies; immunoblotting to detect the non-phospho-counterparts of these proteins was used as the control. GAPDH was used as the loading control. (B) LNCaP cells were treated as described in (A) and immunoblotted using the antibodies as indicated and using GAPDH as the loading control.

Having demonstrated that IL-6 is able to induce autophagy in LNCaP cells in the absence of androgen, we further investigated the potential signaling pathways involved, namely (i) JAK-STAT3, (ii) PI3K-Akt and (iii) MEK-ERK. For these experiments, LNCaP cells were treated with 2.5% CDT or 2.5% CDT plus IL-6. Total cell lysates (TCLs) from these cells were then subjected to immunoblotting with phospho-STAT3, phospho-Akt and phospho-ERK specific antibodies as well as their non-phospho-counterparts. Increased phosphorylation of Akt and STAT3 was only observed after IL-6 treatment (Fig. 9A). ERK phosphorylation was not found to occur after any of the treatments (Fig. 9A). Since Akt is a key positive upstream regulator of mTOR and its activity is increased by phosphorylation, we further analyzed the phosphorylation status of mTOR after treatment. Interestingly, a decreased phosphorylation of mTOR was evident in the 2.5% CDT treated cells and there was an even more significant reduction observed in the 2.5% CDT plus IL-6 treated cells (Fig. 9B). PI3K-Akt is the main positive regulatory pathway for mTOR, which, in turn, negatively regulates autophagy. The down modulation of mTOR by androgen deprivation and androgen deprivation plus IL-6 treatment indicates that the mTOR pathway is not targeted by PI3K-Akt but rather is controlled via a negative regulatory pathway. In order to determine the mechanism that links mTOR inhibition and IL-6 signaling, AMPK, a negative regulator of mTOR, was further analyzed. Consistently with previous findings that have shown there is a small increase in AMPK phosphorylation under androgen deprivation condition in LNCaP cells [48], a slight increase in the amount of phopho-AMPK present was found in 2.5% CDT treated cells (Fig. 9B). Such an increase in phospho-AMPK was also evident when cells were treated with IL-6 plus 2.5% CDT (Fig. 9B). These findings indicate that AMPK activation may responsible for mTOR inhibition when cells are treated with IL-6 under androgen deprivation conditions.

### Downregulation of REST during the IL-6 Induced NED of LNCaP Cells and in Relapsed PCa Specimens

**Figure 10:**
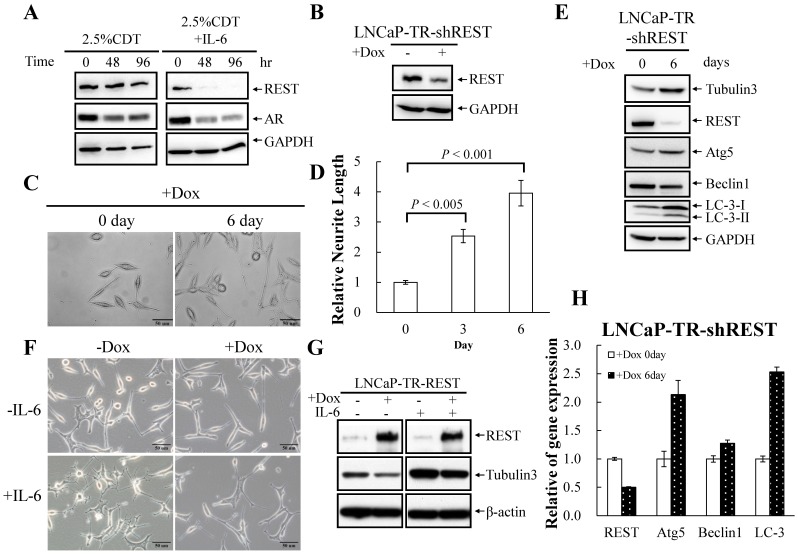
Regulation of NED by REST in LNCaP cells. (A) The level of REST protein declines during IL-6 treatment. LNCaP cells were treated with 100 ng/ml IL-6 for 48 and 96 hours. The expression level of REST was analyzed by immunoblotting using anti-REST antibody. GAPDH was used as the loading control. (B) LNCaP-TR-shREST cells were treated with or without Dox for 48 hours. TCLs were analyzed by immunoblotting using anti-REST antibody. (C) LNCaP-TR-shREST cells were treated with Dox for 6 days. The promotion of neurite outgrowth by REST knockdown was assessed using brightfield microscopy images (40× magnification). (D) The neurite elongation was quantified using the average from 3–5 microscopic fields; *bars*, SD. (E) LNCaP-TR-shREST cells were treated as described in (C). TCLs were prepared and analyzed by immunoblotting using the antibodies as indicated. (F) LNCaP-TR-REST cells were treated with 1 µg/ml Dox in the absence (control) or presence of 100 ng/ml IL-6 for 4 days. Inhibition of IL-6-induced neurite outgrowth by REST overexpression was assessed using brightfield microscopy images (40× magnification). (G) TCLs were obtained from LNCaP-TR-REST cells treated as described in (F); these were then analyzed by immunoblotting using the indicated antibodies. (H) RT-qPCR analysis of total RNA isolated from LNCaP-TR-shREST cells treated as described in (C). The relative mRNA levels of REST, Atg5, beclin1 and LC3 were normalized against GAPDH. Values from three independent data points are reported as mean±S.D.

Next, a cDNA microarray analysis was used to identify IL-6 target genes during autophagy activation and NED. Interestingly, we identified REST as being a neuronal repressor that was decreased in expression in a manner that was inversely coordinated with lineage commitment, which was assessed by measuring the NED markers NSE and tubulin III (Table 1). These results were confirmed by RT-qPCR using LNCaP cells that had undergone IL-6-indued NED (Table 1). REST is a transcriptional repressor that blocks neuronal differentiation [36]. It was also found to inhibit the transcription of the NED marker synaptophysin [39]. A very recent study shows that REST plays an important role in modulating androgen-deprivation induced NED in PCa [40]. All these result support our hypothesis that REST may be another potential mechanism by which NED is induced by IL-6. Consistent with our hypothesis, REST protein was found to be significantly reduced under IL-6 treatment (Fig. 10A). To determine whether REST has a role for NED and autophagy activation, we generated a REST inducible knockdown cell line using LNCaP-TR cells, LNCaP-TR-shREST. Effective knockdown of REST protein in LNCaP cells was identified at 48 hours after Dox treatment (Fig. 10B). Interestingly, the REST knockdown LNCaP cells exhibited remarkableNED. After 6 days of Dox induction, a morphological study showed that the REST knockdown cells displayed a higher degree of NED than control cells (Fig. 10C). Quantification of the results showed that REST knockdown resulted in a significant increase in the neurite length of LNCaP cells (Fig. 10D). Enhancement of cell NED by knockdown of REST was further confirmed by Western blot analysis using tubulin III antibody. Consistent with the cell morphology observations, knockdown of REST increased the expression of tubulin III (Fig. 10E). Interestingly, an increased in the level of LC3 was also induced when REST was silenced. As shown in Figure 10E, both LC3-I and LC3-II were increased by knockdown of REST. This result demonstrates that the autophagy pathway is activated by REST knockdown. To further study whether REST plays a key role in PCa cells NED induced by IL-6, we next generated a REST inducible cell line in LNCaP-TR cells, LNCaP-TR-REST. Effective induction of REST protein in LNCaP cells was identified at 4 days after Dox treatment (Fig. 10G). Consistent with our hypothesis, IL-6 induced neuronal cell morphology changes in LNCaP cells was significantly inhibited when there was REST overexpression (Fig. 10F). Inhibition of cell NED by REST overexpression was further confirmed by Western blot analysis using tubulin III antibody (Fig. 10G).

**Table 1:** A comprehensive RT-qPCR analysis of microarray data of IL-6 treated LNCaP cells.

	Gene Name	RT-qPCR
Neuronal Differentiation	NSE	5.9
	Tubulin III	1.6
	Filamin B	1.6
	RTN1	2.5
	JunB	2.0
	JunD	1.6
	MAP1B	2.3
	REST	−2.5
	AR	−5.0
	ID1	−7.4
Survival	MCL-1	2.0
	IkB	−2.2
	STAT3	1.6
Growth arrest	cdc2	−1.9
	cdc20	−1.8
	cdc6	−3.2
	CyclinA2	−2.5
	CyclinB1	−1.7
	CyclinB2	−1.6
	CyclinD3	−1.4
	CyclinE1	−1.4
	MCM4	−1.8
	RM2	−3.1
	PLK1	−1.6
	Aurora A	−1.6
	Aurora B	−1.6
	RSK90	−2.1
	ASK	−1.7
	GADD45G	2.0
	PML	1.4

Given that REST is a transcriptional repressor that epigenetically suppresses target gene expression, we hypothesized that activation of autophagy by REST knockdown may happen via up-regulation of autophagy genes expression. Therefore we determined the mRNA levels and protein levels of autophagy related genes such as beclin1, Atg5 and LC3 in the REST knockdown LNCaP cells by quantitative RT-PCR and immunoblotting, respectively. We found an increase in the levels of Atg5 mRNA and Atg5 protein expression after REST knockdown for 6 days (Fig. 10H and E). A significant increase in LC3 at both the mRNA level and protein level were also identified after knockdown of REST for 6 days (Fig. 10H and E).

The above findings suggest that the level of REST in cells may be reduced in parallel with the NED process during hormone-refractory relapse. To characterize the correlation between the level of REST and PCa relapse, we examined the expression of REST in our thirteen pairs of primary and hormone-refractory relapsed PCa tissues. Representative IHC results for REST are shown in Figure 6. The IHC-staining results showed that the level of REST is reduced in 62% (8/13) (average REST IR of primary and relapsed PCa tumor was 1.47 and 1.00, respectively; P<0.05) of the relapsed PCa specimen comparing to their primary tumor counterpart. Half of them that have lost REST during relapse show concordant up-regulation of LC3. These findings suggest that down-modulation of REST is part of a relay mechanism for autophagy induction in NE differentiated PCa cells.

## Discussion

Autophagy is an important physiological process that is involved in maintaining the homeostasis of intracellular organelles and thus it helps to prevent carcinogenesis by maintaining cellular integrity and genomic stability. It may also protect against tumorigenesis by promoting autophagic cell death. However, autophagy is a double-edged sword in many cancers; this is because tumor cells are able to hijack the autophagy pathway to promote the cell survival in an environment where there is increased metabolic and other stresses. Autophagy inhibition is therefore an useful treatment route in terms of anti-cancer therapies and is therapeutically beneficial to the above cancers as it sensitizes cancer cells to different chemotherapies [49]. This dual-faced role of autophagy is also observable in PCa. Autophagy acts as a tumor suppressor in the prostate as has been suggested by the observation that *beclin1* gene deletion occurs in many PCas [50]. In contrast, autophagy has also been found to provide survival benefit when PCa cells confront stress; therefore it also promotes tumor progression.

Accumulating evidence suggests that autophagy that is activated by androgen deprivation serves a protective role and might be conducive to the transition to androgen-independence [51], [52]. In addition to androgen-independence, NED is another even more important feature associated with the development of the highly lethal castration-resistant PCa during ADT. PCa-derived NE-like cells are terminally differentiated non-proliferative cells that are highly resistant to chemotherapy. Many studies have identified potential inducers of NED and explored the underline mechanisms. However, no single pathway that is responsible for the development of NED, or is responsible for maintaining the survival of these terminal differentiated NE-like cells, has been identified; such identification might make it a therapeutic target. In this study, we found that autophagy was activated during IL-6 induced NED of PCa cells in the absence of androgen. The activation of the autophagy pathway by IL-6 prompted us to hypothesize that autophagy might be able to account for both the induction of NED as well as the maintaining of survival of these terminally differentiated NE-like cells.

Our findings showed that autophagy is essential for both NED when it is induced by IL-6 under androgen deprivation conditions and the survival of the terminally differentiated NE-like cells. Inhibition of autophagy by CQ severely impaired the NED of LNCaP cells treated with IL-6 (Fig. 3). Further evidence for the essential role of autophagy in NED was obtained using an inducible beclin1 knockdown LNCaP cell line (Fig. 4). Pearson correlation coefficient was used to compute the correlation of expression profiles between IL-6 and various autophagy-related genes using the NCI-60 expression data set (http://discover.nci.nih.gov/cellminer/) and it was found that IL-6 showed a high correlation with one autophagy-related gene, Atg5. Therefore, inducible Atg5 knockdown LNCaP cell lines were also generated as part of this study. Consistent with our hypothesis suggesting that autophagy is essential for NED, the inhibition of autophagy by knocking down Atg5 reduced the degree of NED in LNCaP cells treated with IL-6 significantly (Fig. 5). These findings indicate that the autophagy pathway plays an essential role in NE cell development. These findings were consistent with a recent study showing that IL-6 induced autophagy is responsible for the NED induction by IL-6 in bone metastatic PCa cells [19]. Our hypothesis was further supported by the observation that up-regulation of autophagy genes LC3 occurs in hormone-refractory relapsed PCa tissue samples comparing to their primary tumor counterparts (Fig. 6). Though colocalization of CgA-positive and LC-3-positive cells was not observed, which may be due to the specimens were not used in serial section, the LC3 staining pattern was found to have a similar foci staining pattern to that observed for CgA, which is a marker for PCa NE cells. These findings suggest that autophagy is activated in the NE differentiated cells in relapsed PCa tissue samples.

We also found that autophagy inhibition by CQ induced apoptosis of IL-6 treated LNCaP cells (Fig. 7), which is consistent with cell survival and chemotherapy resistance hypothesis. Moreover, inhibition of autophagy by the independent knockdown of the autophagy-related genes beclin1 and Atg5 was found to potentiate the cytotoxicity of the chemotherapy drug etoposide (Fig. 8). These findings indicate that the autophagy pathway also plays an essential role in maintaining the survival of NE-like cells of PCa tissue. This, together with our findings in autophagy-mediated NED progression, suggest that autophagy inhibition might be attractive as an approach to both the prevention and treatment of the relapsed NE PCa, a subtype of highly aggressive PCa, and as part of NE-related metastatic tumors therapy.

There are many different kinds of NE tumors. NE tumors are epithelial neoplasms that show predominant NED; they arise from the cells of the endocrine/hormonal and nervous systems. In addition to PCa, NED is also a focus for many other tumors that originate from a specific organ, such as gastrointestinal tract, pancreases, lung and breast neoplasms. Such NE tumors frequently are resistant to chemotherapy. The major findings of this study highlight the essentialness of autophagy to IL-6 mediated NED and to the chemoresistance of NE differentiated PCa cells. Taken as a whole, this suggests that autophagy may play a similar role in other types of NE tumors and targeting autophagy may potentially be a useful part of combined therapy when treating a number of different types of NE tumors.

IL-6 activates three major signaling pathways, namely the JAK-STAT3, PIK3-Akt and MEK-ERK pathways. Current knowledge indicates that STAT3 [11], [17] and PI3K [18], [53] activation plays an important role in IL-6 induced NED in PCa cells. The PI3K-Akt pathway has also been found to be critically involved in NED of PCa cells after androgen deprivation [54], [55]. To identify the signaling pathways that are involved in the IL-6-induced activation of autophagy under the androgen deprivation conditions in PCa cells, activation of STAT3, Akt, and ERK was analyzed by immunoblotting using their phospho-specific antibodies. The non-phospho-specific equivalents were included in the immunoblotting as controls. Consistent with previous findings on IL-6 mediated NED, IL-6 treatment in this study was found to induce an increase in phosphorylation of STAT3 but not of ERK (Fig. 9A). However, inhibition of STAT3 activation by AG490 did not block IL-6 induction of autophagy (data not shown). This data is consistent with the findings of Delk *et al* in bone metastatic PCa cells [19]. Akt was also found to be activated by IL-6 (Fig. 9A). Therefore, mTOR, the key negative regulator of the autophagy pathway that is downstream of PI3K-Akt was analyzed by immunoblotting using phospho-specific antibodies. Interestingly, and consistent with autophagy activation, a decrease in mTOR phosphorylation was identified under IL-6 treatment in the absence of androgen (Fig. 9B). Inhibition of mTOR has also been identified as the mechanism involved in autophagy-mediated neuronal cells differentiation [42], [56]. When inhibition of mTOR was carried out in this study, the results were consistent with IL-6 induction of autophagy and the consequential induction of NED in PCa cells via an inhibition of mTOR. However, it is known that mTOR is positively regulated by PI3K-Akt pathway via activation of the receptor tyrosine kinase. Although the PI3K pathway was found to be activated by IL-6 (Fig. 9A), the down-regulation of mTOR activity found in this study indicates that IL-6 regulates mTOR via the activation of a negative regulatory pathway.

**Figure 11:**
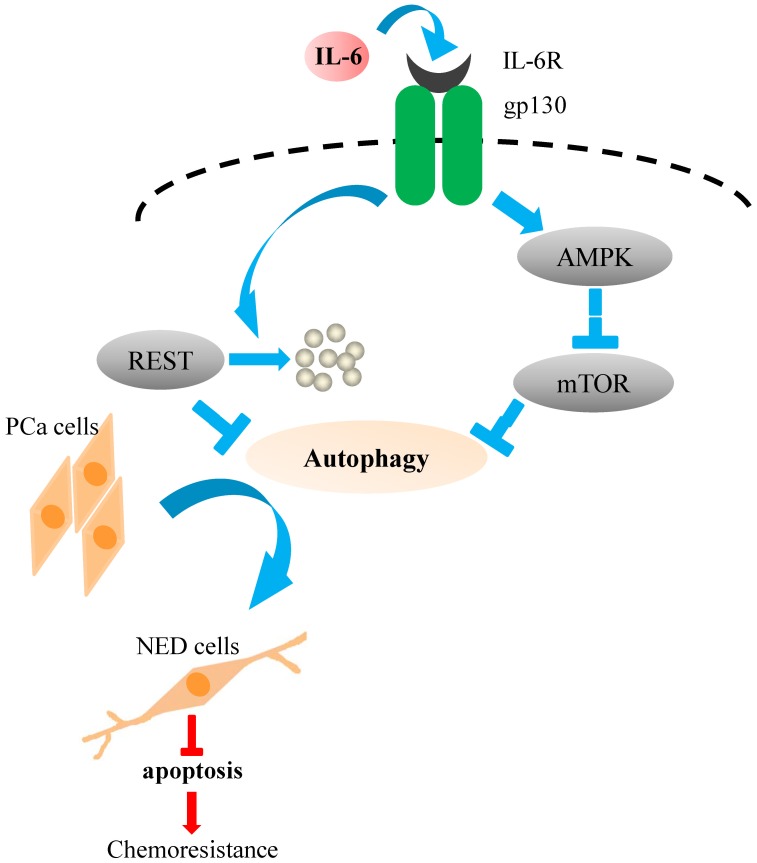
The schematic model of IL-6 regulation of autophagy-dependent NED in PCa cells, LNCaP. Based on our data, autophagy is essential for PCa cells NED and serves a cytoprotective role in NE differentiated PCa cells. IL-6 induces autophagy-dependent NED through activation AMPK/mTOR pathway and down-modulation of REST. Activation of AMPK inhibits mTOR, a key inhibitor of autophagy, which in-turn activates autophagy pathway. Down-modulation of REST level relieves gene silencer REST-mediated transcriptional repression as part of a relay mechanism found in IL-6 induced autophagy.

Currently it is known that mTOR is negatively regulated by AMPK signaling, which is activated by cytokine and metabolic stress. Though AMPK is not the canonical pathway activated by IL-6, it is known to be activated by IL-6 via metabolic regulation [57]–[59]. In addition, another recent study has shown that IL-6 is able to suppress mTOR in a AMPK-dependent and STAT3-independent manner [60]. Moreover, activation of AMPK is involved in the survival of PCa cells that have been subject to stress [48], [61]. Taking the above as a whole, we hypothesized that the suppression of mTOR by IL-6 under the androgen deprivation condition might occur via activation of AMPK pathway. Therefore, levels of phospho-AMPK were analyzed. IL-6 treatment was found to induce a slightly increased in AMPK phosphorylation (Fig. 9B). The fact that the increase in phospho-AMPK is only slight might be a result of the fact that AMPK is already activated in most human PCa cell lines, including LNCaP [62]. The slightly activation of AMPK observed in this study suggests that IL-6 is able to induce a non-canonical pathway, namely AMPK, in order to suppress mTOR, which then activates the autophagy pathway (Fig. 11). However, we cannot exclude the possibility that other pathways are involved in the down-regulation of mTOR during IL-6 treatment under androgen deprivation conditions. Detailed analysis of the mechanisms involved in mTOR inhibition identified in the present study are worthy of further study.

When we analyzed the molecular signature of IL-6 treated LNCaP cells by cDNA microarray analysis, we found a down-regulation of REST transcription (Table 1). Recently, deep RNA sequencing of paired primary and metastatic PCa samples has suggested that the REST transcriptional complex is involved in the development of NE PCa [63]. Moreover, a very recent report showed that REST modulates androgen-deprivation induced NED in PCa cells [40]. Together, these findings suggest that down-modulation of REST by IL-6 may play an essential role in IL-6-induced NED and autophagy identified in this study. Based on this hypothesis, we were able to identify down-regulation of REST protein in IL-6 treated NE differentiated LNCaP cells (Fig. 10A). Tubulin III, which is a NED marker, was found to be increased in expression (Fig. 2C) in parallel with the decrease in the level of REST (Fig. 10A) as the LNCaP cells were induced to undergo NED by IL-6 treatment. To show the functional relevance of REST to PCa NED, we then generated inducible REST knockdown and inducible REST overexpression LNCaP cell lines (Fig. 10B and 10G, respectively). Knockdown of REST in LNCaP cells forced the cells to undergo NED in the absence of the treatment with IL-6 (Fig. 10C and 10D). In contrast, overexpression of REST in LNCaP cells inhibited NED when LNCaP cells were treated with IL-6 (Fig. 10F). Interestingly, autophagy activation (Fig. 10E) and an increase in the transcription of the autophagy genes Atg5 and LC3 (Fig. 10H) was also identified in the REST knockdown cells. This is novel as it is the first time that NED of cancer cells has been shown to utilize the REST pathway for autophagy induction (Fig. 11).
